# Lung-dominant connective tissue disease among patients with interstitial lung disease: prevalence, functional stability, and common extrathoracic features*

**DOI:** 10.1590/S1806-37132015000004443

**Published:** 2015

**Authors:** Daniel Antunes Silva Pereira, Olívia Meira Dias, Guilherme Eler de Almeida, Mariana Sponholz Araujo, Letícia Barbosa Kawano-Dourado, Bruno Guedes Baldi, Ronaldo Adib Kairalla, Carlos Roberto Ribeiro Carvalho

**Affiliations:** University of São Paulo, School of Medicine, Hospital das Clínicas, São Paulo, Brazil. Department of Pulmonology, Instituto do Coração - InCor, Heart Institute - University of São Paulo School of Medicine Hospital das Clínicas, São Paulo, Brazil; University of São Paulo, School of Medicine, Hospital das Clínicas, São Paulo, Brazil. Department of Pulmonology, Instituto do Coração - InCor, Heart Institute - University of São Paulo School of Medicine Hospital das Clínicas, São Paulo, Brazil; University of São Paulo, School of Medicine, Hospital das Clínicas, São Paulo, Brazil. Department of Pulmonology, Instituto do Coração - InCor, Heart Institute - University of São Paulo School of Medicine Hospital das Clínicas, São Paulo, Brazil; University of São Paulo, School of Medicine, Hospital das Clínicas, São Paulo, Brazil. Department of Pulmonology, Instituto do Coração - InCor, Heart Institute - University of São Paulo School of Medicine Hospital das Clínicas, São Paulo, Brazil; University of São Paulo, School of Medicine, Hospital das Clínicas, São Paulo, Brazil. Department of Pulmonology, Instituto do Coração - InCor, Heart Institute - University of São Paulo School of Medicine Hospital das Clínicas, São Paulo, Brazil; University of São Paulo, School of Medicine, Hospital das Clínicas, São Paulo, Brazil. Department of Pulmonology, Instituto do Coração - InCor, Heart Institute - University of São Paulo School of Medicine Hospital das Clínicas, São Paulo, Brazil; University of São Paulo, School of Medicine, Hospital das Clínicas, São Paulo, Brazil. Department of Pulmonology, Instituto do Coração - InCor, Heart Institute - University of São Paulo School of Medicine Hospital das Clínicas, São Paulo, Brazil; University of São Paulo, School of Medicine, Hospital das Clínicas, São Paulo, Brazil, Department of Cardiorespiratory Diseases, University of São Paulo School of Medicine, São Paulo, Brazil; and Director. Department of Pulmonology, Instituto do Coração - InCor, Heart Institute - University of São Paulo School of Medicine Hospital das Clínicas, São Paulo, Brazil

**Keywords:** Idiopathic interstitial pneumonias, Autoantibodies, Connective tissue diseases, Autoimmunity

## Abstract

**OBJECTIVE:**

To describe the characteristics of a cohort of patients with lung-dominant connective tissue disease (LD-CTD).

**METHODS:**

This was a retrospective study of patients with interstitial lung disease (ILD), positive antinuclear antibody (ANA) results (≥ 1/320), with or without specific autoantibodies, and at least one clinical feature suggestive of connective tissue disease (CTD).

**RESULTS:**

Of the 1,998 patients screened, 52 initially met the criteria for a diagnosis of LD-CTD: 37% were male; the mean age at diagnosis was 56 years; and the median follow-up period was 48 months. During follow-up, 8 patients met the criteria for a definitive diagnosis of a CTD. The remaining 44 patients comprised the LD-CTD group, in which the most prevalent extrathoracic features were arthralgia, gastroesophageal reflux disease, and Raynaud's phenomenon. The most prevalent autoantibodies in this group were ANA (89%) and anti-SSA (anti-Ro, 27%). The mean baseline and final FVC was 69.5% and 74.0% of the predicted values, respectively (p > 0.05). Nonspecific interstitial pneumonia and usual interstitial pneumonia patterns were found in 45% and 9% of HRCT scans, respectively; 36% of the scans were unclassifiable. A similar prevalence was noted in histological samples. Diffuse esophageal dilatation was identified in 52% of HRCT scans. Nailfold capillaroscopy was performed in 22 patients; 17 showed a scleroderma pattern.

**CONCLUSIONS:**

In our LD-CTD group, there was predominance of females and the patients showed mild spirometric abnormalities at diagnosis, with differing underlying ILD patterns that were mostly unclassifiable on HRCT and by histology. We found functional stability on follow-up. Esophageal dilatation on HRCT and scleroderma pattern on nailfold capillaroscopy were frequent findings and might come to serve as diagnostic criteria.

## Introduction

There is a dilemma surrounding the classification of patients with interstitial lung diseases (ILDs) and clinical features that are suggestive of *formes frustes* (limited forms) of connective tissue disease (CTD), because such patients do not meet the accepted rheumatological criteria for a definitive diagnosis of CTD.^(^ 1 ^-^ 3 ^)^ Since the first recognition of the nonspecific interstitial pneumonia (NSIP) pattern as a possible independent disease, it has been strongly associated with CTD.^(^ 4 ^)^ Previous studies have shown different characteristics regarding the prognosis and natural history of idiopathic interstitial pneumonia (IIP) with a "rheumatological flavor" but without a definitive diagnosis of CTD.^(^ 5 ^-^ 7 ^)^ The majority of such studies have departed from the NSIP histology to scrutinize the clinical, physiological, and tomographic features of patients. However, it remains unclear whether other ILD patterns are associated with this subgroup, and only a few studies have considered patterns that are either exclusively usual interstitial pneumonia (UIP)^(^ 8 ^,^ 9 ^)^ or mixed.^(^ 6 ^,^ 10 ^)^ Although patients with CTD and ILD have better survival, regardless of their histology,^(^ 11 ^-^ 13 ^)^ uncertainties remain regarding how isolated autoantibody positivity in IIP affects the natural course of the disease and the response to treatment.^(^ 7 ^,^ 14 ^)^

Fischer et al.^(^ 4 ^)^ recently proposed "lung-dominant" CTD, or LD-CTD, as a new classification and the term best suited to describing the association between ILD and undifferentiated CTD, theretofore referred to by myriad terms. The authors proposed comprehensive and restrictive provisional criteria that recognize any classical ILD pattern as a possible association with LD-CTD. Positivity for autoantibodies that are more specific, with special attention to their titers, and histological features that are strongly associated with collagen vascular diseases were also included in the definition of LD-CTD proposed by the authors.^(^ 4 ^)^

We hypothesized that comprehensive and restrictive criteria would be needed in order to define LD-CTD appropriately. We further hypothesized that the definition would be more accurate if ILDs were accompanied not only by autoantibody positivity but also by any extrathoracic feature of CTD. The main objective of this study was to characterize a retrospective cohort of patients in Brazil who met the clinical, functional, serological, tomographic, and histological criteria for a diagnosis of LD-CTD, including the presence of extrathoracic manifestations. We also evaluated how the pulmonary physiology behaves throughout follow-up in patients with LD-CTD.

## Methods

### Patients

**Chart 1 -:**
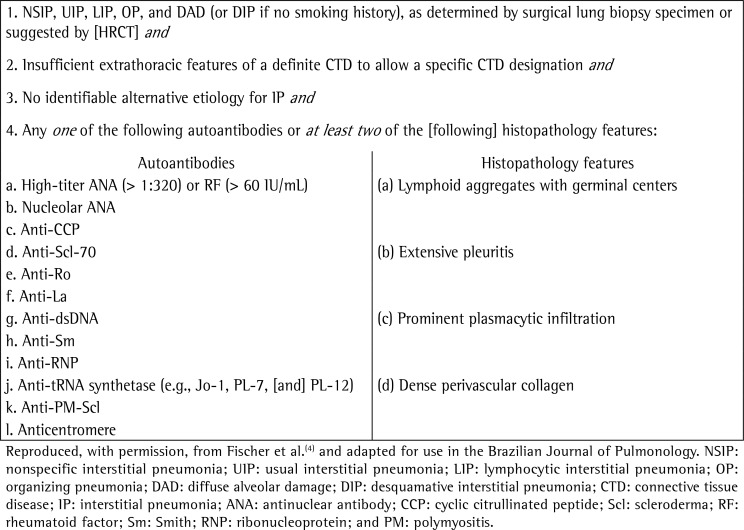
Proposed provisional diagnostic criteria for lung-dominant connective tissue disease.

This was a retrospective study of patients with ILD seen at the outpatient clinic of a tertiary university hospital in Brazil over the previous 16 years (1996-2012). From among the 1,998 cases in the ILD patient database, we selected 75 in which the patients met the LD-CTD criteria proposed by Fischer et al.,^(^ 4 ^)^ as detailed in Chart 1, at the time of their first clinical evaluation. After the records had been evaluated by a multidisciplinary team composed of radiologists, pathologists, and pulmonologists with expertise in the diagnosis of ILD, we excluded patients with classifiable forms of CTD or ILDs with known etiologies, such as hypersensitivity pneumonitis, smoking, and idiopathic pulmonary fibrosis (IPF). In addition, we excluded cases in which basic initial complementary tests were not performed. A rheumatologist also evaluated every case included in the analysis.

### Data collection

On the basis of our review of the patient charts, we selected ILD patients with a high antinuclear antibody (ANA) titer (≥ 1:320), with or without positivity for specific autoantibodies, and at least one clinical extrathoracic feature suggestive of CTD. We collected data related to demographic characteristics; comorbidities; clinical features of CTD (including clinical extrathoracic features at diagnosis and over the course of the disease); imaging findings (HRCT scans of the chest and nailfold capillaroscopy); pulmonary function tests (PFTs) at the initial visit and last available evaluation; biological parameters (including the results of a broad autoantibody panel and routine blood tests); histological features; and details of the medical treatment. 

Within the data collected at the initial evaluation, we searched for extrathoracic features suggestive of CTD, including arthritis; arthralgia; morning stiffness; photosensitivity; cutaneous lesions, such as "mechanic's hands" (hyperkeratosis of the hands or fingers), Gottron's papules, and heliotrope rash; Raynaud's phenomenon; sicca syndrome (dry mouth, dry eyes, or a positive Schirmer's test result); and refractory gastrointestinal symptoms, such as heartburn, acid regurgitation, and dysphagia.

We collected initial and last-registered FVC and FEV_1_ values. We used standard spirometry techniques, and the predicted spirometric values were derived from the data reported for the Brazilian population.^(^ 15 ^)^ The chest HRCT scan patterns were retrieved from radiology reports made by radiologists experienced in evaluating ILD and were based on international consensus criteria.^(^ 16 ^-^ 18 ^)^ To standardize the HRCT criteria for esophageal dilatation on HRCT images, two experienced pulmonologists blindly evaluated HRCT scans for the presence of esophageal dilatation, occurring below the aortic arch with a large (> 10-mm) collection of intraluminal air in the coronal plane on four or more consecutive axial images.^(^ 19 ^)^ The esophagus was also considered dilated when it was filled with fluid or showed an air-fluid level.^(^ 20 ^)^ The autoantibody panel consisted of the following tests: ANA (titer and pattern), rheumatoid factor (RF), anti-cyclic citrullinated peptide (anti-CCP), anti-Ro, anti-La, anti-ribonucleoprotein (anti-RNP), anti-topoisomerase I (anti-Scl-70), anticentromere, anti-Jo-1, anti-DNA, and anti-Smith (anti-Sm). Histological patterns were also retrieved from anatomopathological reports made by experienced lung pathologists and based on international consensus statements.^(^ 16 ^,^ 17 ^)^

### Statistical analysis

Descriptive statistics were obtained with the statistical software STATA, version 12.1 (StataCorp LP, College Station, TX, USA). Categorical variables are expressed as proportions, and continuous variables are expressed as median (interquartile range [IQR]) or mean ± standard deviation. Statistical measurements were performed with the Student's t-test, or Wilcoxon rank sum (Mann-Whitney U) test, for continuous variables, and with Fisher's exact test for categorical variables. To evaluate the associations between two continuous variables, we used multiple linear regression, adjusting for covariates according to model-building strategies. Multiple test correction (Bonferroni correction) was performed for parametric tests. We managed missing data (all missing completely at random) using complete case analysis. The level of statistical significance was set at p < 0.05.

## Results

Of the 75 patients selected, 23 were excluded from the analysis because the spirometric data were incomplete. Therefore, there were 52 patients who met the LD-CTD criteria at baseline (Table 1). Approximately half (28 patients) had a history of smoking, and 21 reported relevant environmental exposure, mainly to mold (13 patients), which was, however, not consistent enough to yield a diagnosis of hypersensitivity pneumonitis. By the end of follow-up-after a median of 61 months (IQR, 48.5-78.0 months)-only 8 patients met the criteria for a definitive diagnosis of a CTD (definite-CTD group): antisynthetase syndrome (n = 3); systemic sclerosis (n = 2); Sjögren's syndrome (n = 2); and rheumatoid arthritis (n = 1). Compared with those who did not meet the criteria for another definitive diagnosis (i.e., the LD-CTD patients), the patients in the definite-CTD group had longer follow-up periods and worse PFT results at the initial evaluation, although the differences were of only marginal statistical significance (Tables 1 and 2). Among the definite-CTD group patients, the histology samples showed only unclassifiable patterns. However, four of those patients underwent transbronchial biopsy. The definite-CTD and LD-CTD groups did not differ statistically in terms of any other characteristics, whether related to the autoantibody profiles or to the extrathoracic features of CTD. 

**Table 1 -:** Baseline characteristics and relevant test results for 52 patients selected from among 1,998 patients with interstitial lung disease seen over a 16-year period.

Variable	Total	LD-CTD	Definite-CTD	p-value
	(n = 52)	(n = 44)	(n = 8)	
Age (years), mean ± SD	56 ± 12	57 ± 12.5	51.5 ± 8.4	NS
Female, n (%)	33 (63)	27 (61)	6 (75)	NS
Follow-up (months), median (IQR)	48 (19-69.5)	30 (16-68)	61 (48.5-78)	0.052
Smoking history, n (%)	28 (53)	25 (56)	3 (37)	NS
Extrathoracic features				
Arthralgia	34 (65%)	29 (66%)	5 (62%)	NS
GERD symptoms	33 (63%)	30 (68%)	3 (37%)	NS
Raynaud’s phenomenon	17 (32%)	14 (32%)	3 (37%)	NS
Skin lesions	16 (30%)	13 (30%)	3 (37%)	NS
Sicca symptoms	12 (23%)	8 (18%)	4 (50%)	0.07
Muscle weakness	13 (25%)	11 (25%)	2 (25%)	NS
Morning stiffness	6 (11%)	5 (11%)	1 (12.5%)	NS
Autoantibody positivity/titer				
ANA, n (%)	44 (84)	39 (89)	5 (62)	0.09
Titer, median (IQR)	1:320 (1:160-1:640)	1:320 (1:160-1:640)	1:320 (1:160-1:320)	NS
High titer (≥ 1:320), n (%)	25 (58)	22 (57)	3 (60)	NS
RF, n (%)	12 (23)	10 (23)	2 (25)	NS
Titer, mean ± SD		1:327 ± 224.5	1:293 ±151	NS
Anti-Ro, n (%)	15 (29)	12 (27)	3 (37)	NS
Anti-La, n (%)	6 (11)	5 (11)	1 (12.5)	NS
Anti-RNP, n (%)	5 (9)	4 (9)	1 (12.5)	NS
Anti-Jo-1, n (%)	6 (11)	4 (9)	2 (25)	NS
Anti-Sm, n (%)	5 (9)	4 (9)	1 (12.5)	NS
Anti-DNA, n (%)	2 (4)	2 (5)	-	NS
Anti-Scl-70, n (%)	1 (2)	1 (3)	-	NS
Anti-CCP, n (%)	2 (4)	1 (3)	1 (12.5)	0.07
Abnormal capillaroscopy, n (%)	17 (32)	15 (34)	2 (25)	NS
Scleroderma pattern, n (%)	13 (25)	11 (25)	2 (25)	NS
HRCT scan pattern	(n = 50)	(n = 42)	(n = 8)	
NSIP, n (%)	22 (44)	19 (45)	3 (37)	NS
UIP, n (%)	6 (12)	4 (9)	2 (25)	NS
Unclassifiable, n (%)	18 (36)	16 (38)	2 (25)	NS
Other, n (%)	4 (8)	3 (7)	1 (12.5)	NS
Esophageal involvement, n (%)	26 (52)	22 (52)	4 (50)	NS
HRCT scan evolution	(n = 40)	(n = 33)	(n = 7)	
Stable, n (%)	22 (55)	19 (57)	3 (42)	NS
Improvement, n (%)	7 (17)	4 (12)	3 (42)	NS
Worsening, n (%)	11 (28)	10 (30)	1 (15)	NS
Histology pattern	(n = 31)	(n = 26)	(n = 5)	
NSIP, n (%)	7 (22)	7 (27)	-	NS
UIP, n (%)	4 (13)	4 (15)	-	NS
Other, n (%)	5 (16)	5 (19)	-	NS
Unclassifiable, n (%)	15 (48)	10 (38)	5 (100)	0.01
Treatment	(n = 52)	(n = 44)	(n = 8)	
None, n (%)	8 (15)	7 (16)	1 (12.5)	NS
Prednisone, n (%)	44 (84)	37 (84)	7 (87)	NS
Prednisone and azathioprine, n (%)	34 (65)	29 (66)	5 (62)	NS

LD-CTD: (patients classified as having) lung-dominant connective tissue disease; Definite-CTD: (patients meeting the criteria for) a definitive diagnosis of a CTD; NS: not significant; IQR: interquartile range; GERD: gastroesophageal reflux disease; ANA: antinuclear antibody; RF: rheumatoid factor; RNP: ribonucleoprotein; Sm: Smith; Scl: scleroderma; CCP: cyclic citrullinated peptide; NSIP: nonspecific interstitial pneumonia; and UIP: usual interstitial pneumonia.

**Table 2 -:** Pulmonary function test results at the initial and final evaluations of 52 patients with interstitial lung disease.

Parameter	Total	LD-CTD	Definite-CTD	p-value
	(n = 52)	(n = 44)	(n = 8)	
Initial evaluation				
FVC (L), mean ± SD	2.10 ± 0.77	2.18 ± 0.76	1.66 ± 0.75	0.09
FVC (% of predicted), mean ± SD	67.5 ± 21.9	69.5 ± 21.5	56.6 ± 22.8	0.08
FEV_1_ (L), mean ± SD	1.79 ± 0.64	1.86 ± 0.63	1.38 ± 0.63	0.08
FEV_1_ (% of predicted), mean ± SD	71.9 ± 23.0	74.0 ± 22.3	58.0 ± 24.4	0.08
Final evaluation				
FVC (L), mean ± SD	2.14 ± 0.77	2.19 ± 0.77	1.88 ± 0.68	0.24
FVC (% of predicted), mean ± SD	71.8 ± 22.3	74.0 ± 22.0	61.2 ± 22.2	0.14
FEV_1_ (L), mean ± SD	1.74 ± 0.56	1.78 ± 0.56	1.49 ± 0.57	0.28
FEV_1_ (% of predicted), mean ± SD	72.9 ± 22.0	75.3 ± 21.9	60.0 ± 24.4	0.07

LD-CTD: (patients classified as having) lung-dominant connective tissue disease; and Definite-CTD: (patients meeting the criteria for) a definitive diagnosis of a CTD.

In the LD-CTD group (n = 44), there was a predominance of females and the median follow-up period was 30 months (IQR, 16-68 months), as shown in Table 1. The shortest follow-up period was 6 months and the longest was 120 months. As can also be seen in Table 1, the most prevalent extrathoracic features were arthralgia and symptoms of gastroesophageal reflux disease (GERD), both in approximately two-thirds of the group, followed by Raynaud's phenomenon, in 14 patients (32%); cutaneous lesions, in 13 patients (30%); and proximal muscle weakness, in 11 patients (25%). 

We found that, in the LD-CTD group, the most prevalent autoantibody was ANA, which was identified in 39 patients, with a median titer of 1:320 (IQR, 1:160-1:640), followed by anti-Ro, in 12 patients, and RF, in 10 (Table 1). However, we did not classify these patients as having LD-CTD solely on the basis of ANA positivity. A diagnosis of LD-CTD can be made on the basis of a high titer for any autoantibody. Therefore, some patients were included in the LD-CTD group because they had a high titer for a specific autoantibody despite showing a low ANA titer. A high ANA titer (≥ 1:320) was identified in 22 of the 39 ANA-positive patients, and the remaining 17 ANA-positive patients were included in the LD-CTD group on the basis of high titers for anti-Ro (5 patients), RF (4 patients), anti-Sm (3 patients), anti-Jo-1 (2 patients), anti-La (2 patients), or anti-DNA (1 patient).

Nailfold capillaroscopy was initially performed in 22 patients, two of whom later received a definitive diagnosis of a CTD. Both had a scleroderma pattern of capillary changes and eventually met the criteria for systemic sclerosis. In the LD-CTD group, capillaroscopic abnormalities were identified in 15 patients, a scleroderma pattern being seen in 11 (Table 1).

**Figure 1 -:**
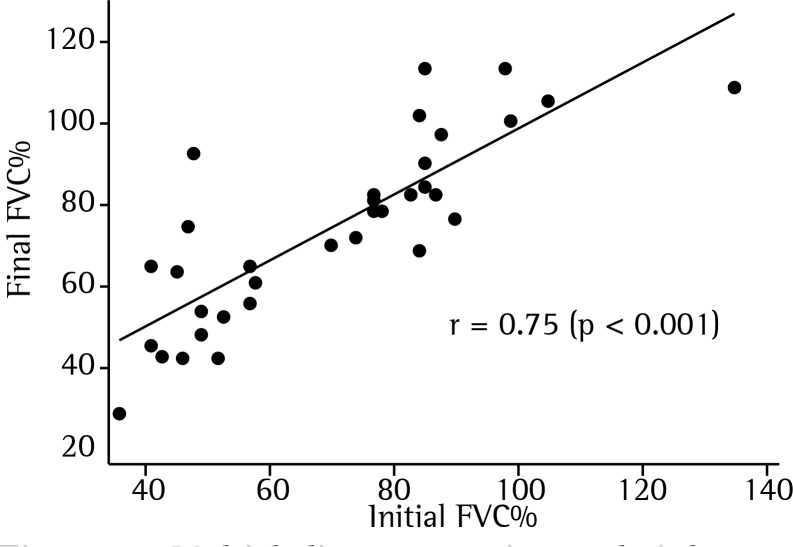
Multiple linear regression analysis between initial and final FVC as a percentage of the predicted value (FVC%) in patients with lung-dominant connective tissue disease (n = 32), showing a strong correlation between initial and final values after adjustment for covariates (time between measures, age, gender, treatment, and antinuclear antibody titer).

At diagnosis, the PFT results for the LD-CTD group patients showed mild restriction, as evidenced by low mean FVC-as a percentage of the predicted value (FVC%, 69.5% ± 21.0%) or as an absolute value (2.18 ± 0.76 L)-with no obstructive pattern (Table 2). After a median follow-up period of 30 months (IQR, 15-57 months), there were no clinically or statistically significant changes in the mean FVC (74% ± 22%; 2.19 ± 0.77 L). As can be seen in Figure 1, multiple linear regression analysis comparing the initial and final FVC% revealed that, even after adjustments for age, gender, treatment, interval between measures, and ANA titer, the initial values were the main predictors of final values (r2 = 0.75; p < 0.001). Subjects who met the criteria for a definitive diagnosis of CTD tended to have worse physiology at initial evaluation, although the difference was of only marginal statistical significance (Table 2).

**Figure 2 -:**
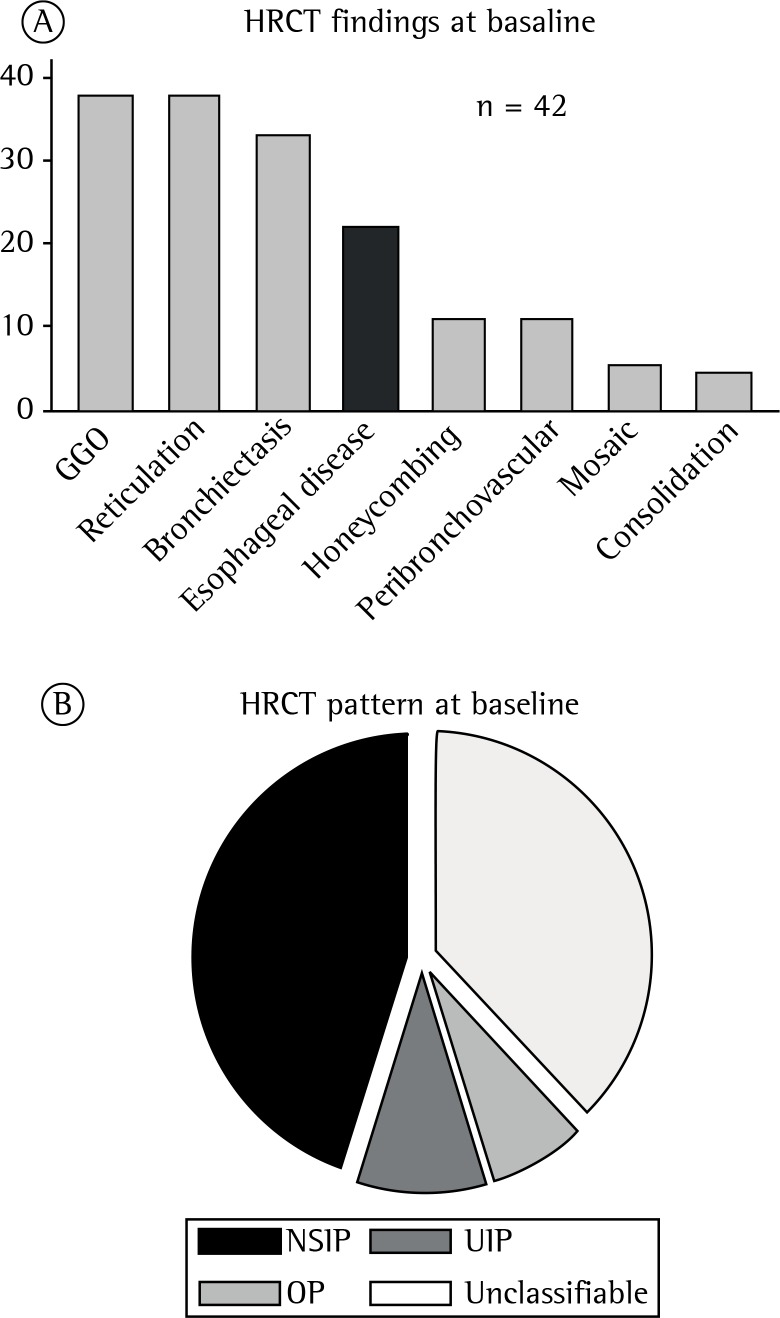
First-evaluation chest HRCT scan findings in patients with lung-dominant connective tissue disease. (A) Bar chart of the total count of HRCT findings in 42 patients. Although ground-glass opacity (GGO), reticulation, and traction bronchiectasis predominated, roughly half of the scans met the published criteria for esophageal disease.(20) (B) Pie chart of the patterns assigned by an experienced radiologist. NSIP: nonspecific interstitial pneumonia; UIP: usual interstitial pneumonia; and OP: organizing pneumonia.

First-evaluation HRCT scans of the chest were available for 42 patients. As depicted in Figure 2, the predominant patterns were ground-glass opacities (in 90%), reticulation (in 90%), and traction bronchiectasis (in 78%). Twenty-two patients showed diffuse esophageal dilatation. Peribronchovascular distribution was reported in 11 patients, even among those with deemed-definite classical HRCT patterns. The HRCT pattern was classifiable in 26 patients, NSIP and UIP patterns being noted in 19 and 4 patients, respectively. Most importantly, 36% of the initial HRCT scans were considered unclassifiable after evaluation by an experienced thoracic radiologist. Follow-up HRCT scans were available for only 33 patients, and the findings remained unchanged in 57%. 

Histological data were available for 26 of the LD-CTD group patients. There were 18 surgical samples and 8 transbronchial samples, evaluation of the latter being conclusive in only one patient with organizing pneumonia. An unclassifiable pattern, mainly transbronchial, was seen in 38% of the samples. The predominant pattern in the surgical samples was NSIP (in 27%), followed by UIP (in 15%) and organizing pneumonia (in 16%). Immunosuppressant therapy had been started in 44 of the LD-CTD group patients, and all of them received prednisone. The treatment regimen comprised the azathioprine/prednisone combination in 66% of the cases.

## Discussion

We have reported the findings of a retrospective evaluation of a group of LD-CTD patients characterized by a predominance of young female patients who complained mainly of arthralgia and GERD symptoms, showed mild restriction on PFTs, were physiologically stable on follow-up, and predominantly had an NSIP pattern (tomographically and histologically). The most relevant autoantibodies in our sample were ANA, anti-Ro, and RF. A scleroderma pattern of capillary changes was observed in 75% of the patients who underwent nailfold capillaroscopy, and diffuse esophageal dilatation was seen on half of the HRCT scans of the chest. Both findings support the relevance of the "rheumatological flavor" in such cases. From a large population of patients diagnosed with ILD, only approximately 2.6% initially met the criteria for an LD-CTD etiology. To date, there has been no estimate of the prevalence of LD-CTD in such a population. It is of note that, after a reasonable follow-up period, 15% of those patients prospectively met the criteria for a definitive diagnosis of a CTD. 

There is growing recognition that ILD might be the first or an isolated manifestation of a CTD. ^(^ 21 ^)^ In 1995, Homma et al.,^(^ 22 ^)^ having followed, for up to 11 years, 68 patients in whom the initial evaluation showed no clinical or serological evidence of CTD,^(^ 1 ^)^ showed that it is possible for ILD to be the sole presentation of occult CTD. Homma et al.^(^ 22 ^)^ showed that the incidence of definite CTD during long-term follow-up was 19%, similar to the rate observed in the present study, and also found no difference between patients who have definite CTD and LD-CTD patients from a clinical or serological standpoint. The authors concluded that there are no clinical markers that are useful in predicting which patients will develop a definite CTD. 

Since Homma et al.^(^ 22 ^)^ first suggested that ILD could be the pulmonary manifestation of an undefined systemic autoimmune disease, the body of literature on the subject has grown considerably. Kinder et al.^(^ 5 ^)^ sought to determine whether idiopathic NSIP is actually the pulmonary manifestation of a systemic autoimmune disease and, consequently, the respiratory counterpart of what rheumatologists know as undifferentiated CTD (UCTD).^(^ 23 ^)^ By applying a broader set of UCTD criteria, the authors compared UCTD patients with IIP patients (specifically IPF patients) and concluded that such criteria could be used in predicting NSIP.^(^ 5 ^)^ These preliminary results were followed by those from several retrospective cohort studies employing different diagnostic criteria and terminology to refer to ILD patients with equivocal CTD features. Corte et al.^(^ 7 ^)^ called into question the specificity of UCTD criteria in predicting NSIP and suggested that only specific features such as Raynaud's phenomenon and a compatible demographic profile (female < 50 years of age) could predict an NSIP pattern in such patients. Therefore, the authors of some studies have applied inclusion criteria that are more stringent. One such study was conducted by Vij et al.,^(^ 6 ^)^ who thus defined an entity referred to as autoimmune-featured ILD. The authors described a UIP-predominant group of patients, in which UIP was identified on the basis of CT scans in 62% and histology in 81%, with characteristics similar to those of patients with IPF (older and male) and, as in the present study, relevant prevalence of GERD symptoms. However, their findings contrast with ours regarding ILD pattern prevalence, because we recognize the relevance of the unclassifiable pattern to this population. Patients with unclassifiable IIP have only recently come to be considered a possible distinct subgroup of ILD patients. When a patient cannot be satisfactorily classified, a diagnosis of unclassifiable IIP is suggested, which is an acknowledgment that a final diagnosis might not be achieved after a multidisciplinary discussion. ^(^ 17 ^,^ 24 ^)^ Another retrospective study predicted a 10% prevalence of unclassifiable cases in a large ILD cohort that was characteristically heterogeneous in terms of several clinical variables.^(^ 24 ^)^ It is noteworthy that 70% of our final pathological unclassifiable patterns were observed in transbronchial biopsy samples, which is in accordance with the findings of Ryerson et al.,^(^ 24 ^)^ who reported that 69% of the unclassifiable cases identified in their study were attributable to biopsy samples being either insufficient or unavailable. Our results embody recognition of the updated (2013) American Thoracic Society/European Respiratory Society joint statement on multidisciplinary classification of ILD, which states that unclassifiable interstitial pneumonia often proves to be related to CTD, especially when there is an overlap of histological patterns within the same sample.^(^ 17 ^)^ Even though transbronchial biopsy plays an unequivocal role in the histological classification of ILD, the limitations of transbronchial biopsy samples should be acknowledged.^(^ 25 ^)^

When we excluded the patients who had definite CTD, we found a strong correlation between initial and final FVC%, suggesting that the main contributor to the final value is the initial measurement itself (Figure 1). After adjusting for several covariates, we found that PFTs remained stable throughout follow-up. Despite the inconclusive role played by histological patterns and the possibility of multiple patterns in LD-CTD patients, the stability of lung function indicates that the disease was milder in our sample than in historical samples of patients with IPF.^(^ 11 ^)^ Such stability was also noted on HRCT scans of the chest: at the last evaluation, 55% of available scans showed stable interstitial abnormalities. In contrast with our finding of stable PFT results, Kinder et al.^(^ 26 ^)^ reported significantly greater improvement in FVC% during follow-up in patients with UCTD than in those with IPF. However, the authors defined improvement at a low cutoff (an absolute increase ≥ 5%), lung function remained stable in a third of their sample, and the diagnostic criteria employed in their study differed from those applied in our study.

Nailfold capillaroscopy is an easily implemented, noninvasive methodology for evaluating the microvascular abnormalities commonly found in several types of CTDs, especially systemic sclerosis, polymyositis/dermatomyositis, and mixed CTD. ^(^ 27 ^)^ In our sample, 22 patients were submitted to nailfold capillaroscopy, of whom 17 were found to have at least one significant microvascular abnormality, substantiating the suspicion of CTD. The sensitivity and specificity of nailfold capillaroscopy have yet to be evaluated. However, in the recent American College of Rheumatology/New European League Against Rheumatism revised classification criteria for systemic sclerosis, capillaroscopy is given only minor weight in a probability score-notably, the same weight given to interstitial lung abnormalities. ^(^ 28 ^)^ Therefore, microvascular alterations are still under scrutiny as predictors of definite CTD.^(^ 27 ^)^ We strongly recommend that, in association with close physical examination of the hands, nailfold capillaroscopy be included in the armamentarium for the initial evaluation of ILD patients, mainly in those under high suspicion of having CTD, with specific autoantibody positivity, and presenting with overt lesions of the skin or joints of the hands. Another easily identifiable characteristic suggestive of associated systemic autoimmunity is esophageal dilatation on HRCT scans, recognizable by a large collection of intraluminal air that is fluid-filled or has an air-fluid level.^(^ 20 ^)^ In the present study, we observed a 50% prevalence of esophageal impairment, including hiatal hernia and esophageal dilatation, corroborating our high frequency of GERD symptoms and suggesting that it is important to screen for esophageal dysmotility when a CTD etiology is suspected.

Our study has certain limitations, in particular the retrospective design, which accounted for a considerable amount of missing data-an obstacle that we overcame by performing a complete case-analysis. In addition, the evaluation time points varied widely among patients, reducing the external validity of our results and possibly contributing to an overestimation of physiological stability and an underestimation of the odds of developing a definite CTD. Follow-up was longer in the definite-CTD group patients than in the LD-CTD group patients, and the difference was statistically significant, albeit only marginally so (p = 0.052). Furthermore, in the majority of patients in our sample, diffusion of carbon dioxide was not measured. However, it can be argued that, despite the ongoing debate on the appropriateness of FVC as a surrogate marker for disease evolution, it is a physiological measure that is frequently used as such.^(^ 26 ^,^ 29 ^)^ Although evidence of esophageal impairment was common in our sample, that should be interpreted with caution, because our findings (which were based on imaging and symptoms) were only suggestive of esophageal involvement (specific tests to identify unequivocal esophageal disease were not conducted). Nevertheless, this retrospective study was the first of its kind to include patients from Brazil, where economic status might play a role in delaying diagnosis and healthcare access. Moreover, we used a more stringent criterion as a starting point for cohort definition,^(^ 4 ^)^ allowing many different classical ILD patterns to be included in the study. To our knowledge, ours is the first study assessing the importance of evaluating nailfold capillaries for the diagnosis of LD-CTD.

In summary, a fair number of patients with ILD might present with one or more features of a CTD without meeting the established diagnostic criteria for such. Among such patients, lung disease seems to be characteristically mild, functional stability being the main feature. Attention to the standard extrathoracic features of CTD should be accompanied by attention to less straightforward GERD symptoms, diffuse esophageal dilatation on HRCT scans, and specific capillaroscopy findings. Although much, if not all, of the current evidence related to LD-CTD relies on retrospective designs, the importance of prospective studies involving LD-CTD patients cannot be overstated. In addition, the relevance of subgrouping patients with ILD into idiopathic, definite-CTD, and LD-CTD groups should be evaluated more comprehensively, specifically regarding prognosis and treatment response. Otherwise, this classification will lack translational use in real-life situations.
